# Evaluation of the analytical anisotropic algorithm in an extreme water–lung interface phantom using Monte Carlo dose calculations

**DOI:** 10.1120/jacmp.v8i1.2324

**Published:** 2007-02-28

**Authors:** Isabelle Marie Gagné, Sergei Zavgorodni

**Affiliations:** ^1^ BC Cancer Agency, Department of Medical Physics Vancouver Island Centre Victoria British Columbia Canada; ^2^ University of Victoria Department of Physics and Astronomy Victoria British Columbia Canada

**Keywords:** lung inhomogeneity correction, Monte Carlo calculations, analytical anisotropic algorithm, pencil beam convolution, AAA, PBC

## Abstract

Our study compares the performance of the analytical anisotropic algorithm (AAA), a new superposition–convolution algorithm recently implemented in the Eclipse (Varian Medical Systems, Palo Alto, CA) Integrated Treatment Planning System (TPS), to that of the pencil beam convolution (PBC) algorithm in an extreme (C‐shaped, horizontal and vertical boundaries) water–lung interface phantom. Monte Carlo (MC) calculated dose distributions for a variety of clinical beam configurations at nominal energies of 6‐MV and 18‐MV are used as benchmarks in the comparison. Dose profiles extracted at three depths (4, 10, and 16 cm), two‐dimensional (2D) maps of the dose differences, and dose difference statistics are used to quantify the accuracy of both photon‐dose calculation algorithms. Results show that the AAA is considerably more accurate than the PBC, with the standard deviation of the dose differences within a region encompassing the lung block reduced by a factor of 2 and more. Confidence limits with the AAA were 4% or less for all beam configurations investigated; with the PBC, confidence limits ranged from 3.5% to 11.2%. Finally, AAA calculations for the small 4×4 18‐MV beam, which is poorly modeled by PBC (dose differences as high as 16.1%), provided the same accuracy as the PBC model of the 6‐MV beams commonly acceptable in clinical situations.

PACS number: 87.53.Bn

## I. INTRODUCTION

To achieve tumor control and minimize normal‐tissue complications, radical radiotherapy requires an accuracy of 5% in the delivery of absorbed dose.^(^ ^1^ ^,^ ^2^ ^)^ This requirement implies that no more than 2%–3% uncertainty is allowed in the treatment planning calculations. That demand is extremely challenging when heterogeneous tissues such as lung and bone are involved.^(3)^ Currently, the “gold standard” for three‐dimensional (3D) dose calculations is the Monte Carlo (MC) simulation, in which electron and photon transports in materials are modeled using probability distributions.^(^ ^4^ ^–^ ^8^ ^)^

Superposition–convolution algorithms are arguably the most accurate algorithms commonly available in commercial systems.^(^ ^9^ ^–^ ^12^ ^)^ These algorithms compute the dose in the patient as the superposition of the total energy released per unit mass (“terma”) with an energy deposition kernel that represents the spread of energy from the primary photon interaction site throughout the volume.

A new analytical anisotropic algorithm (AAA) for photon dose calculations, based on the superposition–convolution method, has been recently developed^(^ ^13^ ^–^ ^15^ ^)^ and implemented in the Eclipse (Varian Medical Systems, Palo Alto, CA) Integrated Treatment Planning System (TPS).^(16)^ Unlike the pencil beam convolution (PBC) algorithm, the AAA employs spatially‐variant MC‐derived convolution scatter kernels and has separate modeling for primary photons, scattered extra‐focal photons, and contaminant electrons. Tissue inhomogeneities are accounted for anisotropically in the 3D neighborhood through the use of radiologic scaling of the dose deposition functions in the beamlet direction and electron‐density‐based scaling of the photon scatter kernels in 16 lateral directions.^(16)^ The final dose distribution is obtained by superposition of the doses from the photon and electron convolutions.

Numerous approaches exist to verify and compare the accuracy of dose calculation algorithms,^(17)^ the most common being point dose, one‐dimensional (1D) profile, and two‐dimensional (2D) isodose line comparison with experimental measurements.^(^ ^12^ ^,^ ^18^ ^–^ ^21^ ^)^ Because of the difficulties associated with the experimental measurements in complex geometries, many investigators are currently using the MC technique to evaluate the accuracy of modern 3D radiotherapy TPS algorithms.^(^ ^4^ ^,^ ^5^ ^,^ ^9^ ^,^ ^10^ ^,^ ^22^ ^,^ ^23^ ^)^ However, most studies involving MC verification of the treatment planning algorithms rely on 1D line and qualitative 2D isodose line comparisons.

Fogliata et al.^(24)^ recently used an extensive set of measurements to investigate the performance of the AAA as compared with the PBC in water. They found that the AAA calculations reproduced the measured data satisfactorily for all open and wedged beams investigated. On average, differences of less than 1% or 1 mm were reported for the percentage depth dose curves. In addition, they found that dose profiles in the flattened region deviated by less than 1%.

The present study compares the accuracy of the AAA (version 7.5.0.7, implemented in the Eclipse TPS) and the PBC (version 7.2.34.0) in an extreme water–lung interface (EWLI) phantom^(23)^ using MC‐calculated 2D dose distributions as benchmarks. The phantom, having three water–lung interfaces (two horizontal and one vertical), presents very challenging conditions for treatment‐planning dose calculation algorithms. Cranmer‐Sargison et al.^(23)^ demonstrated previously that the PBC over‐predicted the dose to the lung portion of the phantom by ~15% in the beam penumbra region. Our study evaluates the performance of both the PBC and the AAA in this phantom and particularly aims to investigate if the newly implemented AAA rectifies the problems previously reported by Cranmer‐Sargison et al.^(23)^

## II. METHODS AND MATERIALS

### A. PBC and AAA

The PBC^(^ ^25^ ^–^ ^27^ ^)^ and AAA^(^ ^13^ ^–^ ^16^ ^,^ ^24^ ^)^ treatment planning algorithms used in the present work are well described in the literature. This section only summarizes the two.

In the PBC, the dose *D*(*x,y,z*) deposited at a point by a therapeutic field *F* of photons is calculated as a convolution of energy fluence Ψ or *terma* with respective dose deposition kernel $K_{w}$ pre‐calculated for a narrow (“pencil”) beam in water:

(1) $$D(x,y,z)=\iint_{F}\Psi (x^{\prime },y^{\prime })\frac{K_{w}(x-x^{\prime },y-y^{\prime },z)}{\rho }dx^{\prime }dy^{\prime }.$$

The depth *z* of the dose deposition is scaled with media density ρ, but the dose kernel is invariant laterally.

The AAA is also convolution based, with the dose from each pencil beam (beamlet) being calculated through a convolution. The beamlet energy fluence is separated into components from primary photons, extrafocal photons, and contaminant electrons originating mainly in the flattening filter, ion chamber, collimating jaws, and air. The dose contribution $D_{\beta }(x,y,z)$ from beamlet β is modeled through convolution of its fluence Φ and energy deposition density function *I*(*z*,ρ) with scatter kernel *K*(*x,y,z*,ρ), that defines the lateral dose scattering in the phantom(^16^):

(2) $$D_{\beta }(x,y,z)=\Phi_{\beta }\times I_{\beta }(z,\rho )\times \iint_{\beta }K_{\beta }(x^{\prime }-x,y^{\prime }-y,z,\rho )dx^{\prime }dy^{\prime }.$$

Each contributing function (fluence, energy deposition density function, and scatter kernel) is defined separately for each of the energy fluence components. Functions representing the energy fluence components and the primary and scatter kernels are expressed analytically, and the convolution integral^(2)^ over the beamlet dimensions has also been solved analytically. That is why the algorithm is termed “analytical.” The feature of the AAA that distinguishes it from the PBC is that the scatter kernels are density dependent and are evaluated in multiple directions laterally from the beamlet. In addition, the photon scatter is convolved with a density‐scaled kernel along the beamlet direction to more accurately reproduce the dose at the border of heterogeneities. The total dose *D*(*x,y,z*) deposited at a point by a therapeutic beam is calculated as superposition of beamlet contributions $D_{\beta }(x,y,z)$.

### B. TPS calculations

**Figure 1:**
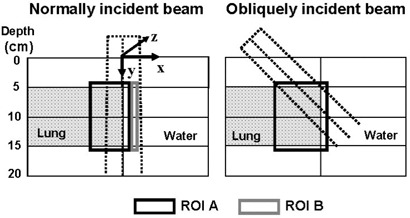
Cross‐sectional view of the extreme water–lung interface phantom and the two regions of interest (ROIs) used to quantitatively assess the performance of the treatment planning system algorithm in this complex geometry. Shown are the ROIs with respect to the 4×4 cm normally and obliquely incident beams

We used the Eclipse TPS to compute the dose distributions for a number of open beam configurations (see Table 1) with a source‐to‐surface distance of 100 cm in our EWLI phantom. The EWLI phantom,^(^ ^23^ ^)^ which consists of two 5‐cm‐thick blocks of lung material surrounded by solid water on three sides (see Fig. 1), presents a challenge for most commercial algorithms because of its sharp vertical and horizontal boundaries. We investigated two photon dose calculation models supported by the Eclipse Integrated TPS—the AAA (version 7.5.0.7) and the PBC (version 7.2.34.0)—in combination with the modified Batho inhomogeneity correction. The TPS calculations were performed on the heterogeneous phantom computed tomography data set, using a 0.25‐cm grid size and normalized to 100% as per Table 1. Normal and oblique beams were modeled to approximate lung dosimetry in mediastinum and breast treatments.

**Table 1:** Open beam configurations used in both the Monte Carlo and treatment planning system calculations, and physical location where the dose distributions were normalized to 100%

Beam configuration (MV)	Beam energy (cm)	Field size (degrees)^(a)^	Gantry angle (*x,y,z*)	Normalization point
1	6	10×10	0	(2.5, 1.5, 0)
2	6	4×4	0	(1.0, 1.5, 0)
3	6	4×4	315^(a)^	(−5.0, 1.5, 0)
4	18	10×10	0	(2.5, 3.5, 0)
5	18	4×4	0	(1.0, 3.5, 0)
6	18	4×4	315^(a)^	(−3.0, 3.5, 0)

(a) The central axis of the normally incident beams was located at the vertical lung–water interface. For the obliquely incident beams, the point of entry was 7.5 cm left of the interface

### C. MC calculations

**Figure 2:**
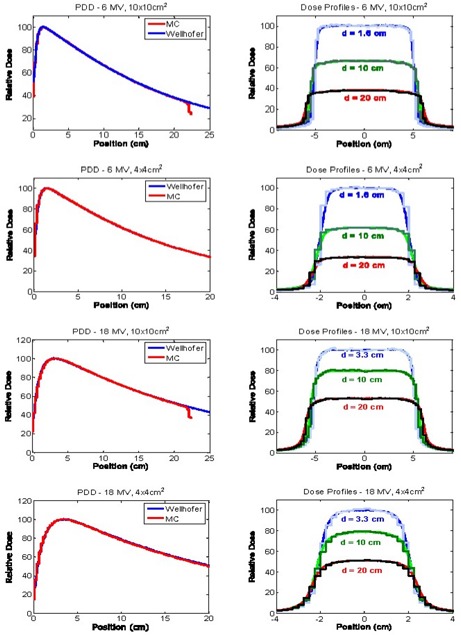
Percentage depth–dose (PDD) curves and beam profiles for the 6 MV and 18 MV 10×10 cm and 4×4 cm fields, measured in water and modeled using our Monte Carlo system

We used the BEAMnrc and DOSXYZnrc^(^ ^28^ ^,^ ^29^ ^)^ radiation transport codes to carry out MC calculations for the same EWLI phantom and beam configurations as were used for the TPS calculations. The MC code modeling the 6‐MV and 18‐MV photon beams for the Varian 21EX linear accelerator used in our investigation has been described in detail by Cranmer‐Sargison et al.^(^ ^23^ ^)^ and tested for open fields both on‐ and off‐axis. Since the publication by Cranmer‐Sargison et al.^(23)^, this model has been further improved to provide closer agreement between MC calculations and the measured data, as shown in Fig. 2 for the beam configurations used in our study. Particle transport parameters were set as follows:

- AE=ECUT=0.700 MeV,
- AP=PCUT=0.010 MeV, and
- ESAVE=2.0 MeV in BEAM modeling.^(28)^

Dose distributions were calculated using a voxel size of 0.25×0.25×2 cm and 0.25×0.25×1 cm for the 10×10‐cm and 4×4‐cm field sizes respectively and normalized to 100% as per Table 1. The coordinate system used in the calculations is shown in Fig. 1, demonstrating that the long side of the voxel was in the nongradient direction of the dose distribution and in the nonvariant direction of the phantom. For each open beam configuration, a total of $5.0\times 10^{8}$ particles were transported into the phantom by recycling and redistributing^(29)^ each particle in the phase space file containing 65 million particles. Particles were recycled less than fifty times, and restarting of the phase space was avoided. The resulting average MC dose uncertainty was less than 1%.

### D. Verification of TPS calculations: comparison with MC

Verification of the TPS‐calculated dose distributions was performed by extracting beam profiles^(^ ^21^ ^,^ ^23^ ^,^ ^30^ ^)^ at three depths (4, 10, and 16 cm) from 3D distributions and comparing them with the MC profiles. Also, in the present study, 2D distributions of the dose difference $\Delta D=D_{\text{TPS}}-D_{\text{MC}}$ were used to evaluate the accuracy of the PBC and the AAA in a heterogeneous phantom. For each open beam configuration, axial 2D dose difference distributions (passing through the centre of the open beam) were obtained in MATLAB (version 7.0, release 14, May 2004: Mathworks, Natick, MA) by subtracting the MC doses from those calculated with the PBC or the AAA.

Two rectangular regions of interest (ROIs) were outlined in the phantom for further quantitative analysis, one region encompassing the lung inhomogeneity and surrounding solid water (ROI A) and the other being a region distant from the inhomogeneity and beyond the buildup area (ROI B). Fig. 1 depicts, for both the normally and obliquely incident beams, the ROIs superimposed on the EWLI phantom cross‐section. Metrics such as mean difference, standard deviation of the differences, and confidence limits (|mean|+1.5 standard deviation) ^(^ ^31^ ^,^ ^32^ ^)^ were computed for both ROIs.

## III. RESULTS AND DISCUSSION

**Figure 3:**
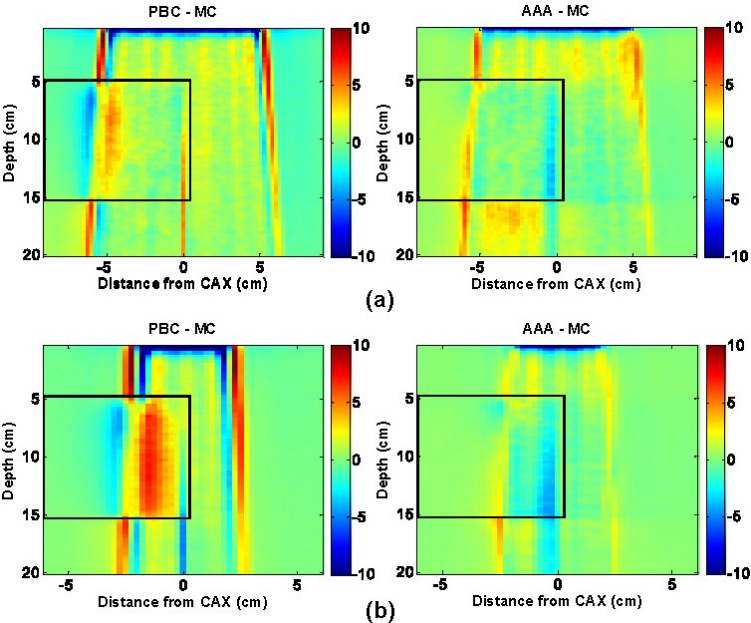
Pencil beam convolution (PBC)–Monte Carlo (MC) and analytical anisotropic algorithm (AAA)–MC dose difference maps for 6‐MV, normal‐incidence beam configurations, where CAX is the central axis: (a) 10×10 cm, 0‐degree gantry angle; and (b) 4×4 cm, 0‐degree gantry angle

**Figure 4:**
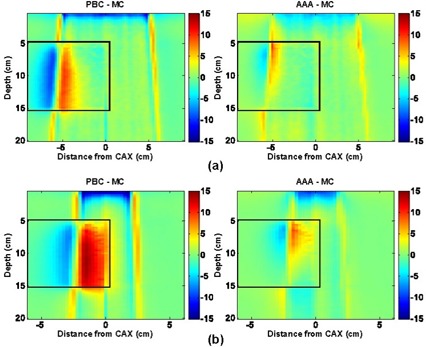
Pencil beam convolution (PBC)–Monte Carlo (MC) and analytical anisotropic algorithm (AAA)–MC dose difference maps for 18‐MV, normal‐incident beam configurations, where CAX is the central axis: (a) 10×10 cm, 0‐degree gantry angle; and (b) 4×4 cm, 0‐degree gantry angle

Figs. 3 and 4 show the dose difference distributions for the 6‐MV and 18‐MV normal incident beam configurations as scaled color maps. For the 6‐MV beams, the minimum dose difference of −10% and the maximum dose difference of +10% are assigned to dark blue and dark red colors respectively. For the 18‐MV beams, the minimum and maximum dose difference values given to dark blue and dark red are −15% and +15% respectively.

Dose differences on the water half (right side) of the EWLI phantom demonstrate the capability of each algorithm to reproduce beam attenuation and profiles in homogeneous media—and beam penumbra. One can see that the PBC and the AAA both properly reproduce the dose in water (dose differences within ROI B were all less than ±2%) when electronic equilibrium conditions prevail. Both algorithms also estimate the penumbral dose to within ±2 mm of the MC dose. However, distances to agreement in the penumbra region were, on average, three times smaller for the AAA (±0.5 mm) than for the PBC (±1.5 mm). Therefore, for the 4×4‐cm and 10×10‐cm field sizes alike, the AAA demonstrates superior penumbra modeling with very small deviations from MC‐modeled penumbra. This finding agrees with the measurements in water reported by Fogliata et al.^(24)^

The light‐yellow stripes visible in the dose difference maps for both algorithms are attributable to latent uncertainty^(33)^ of up to 1% in the BEAM phase space. This uncertainty arises from the fact that the phase space commonly used as a source in MC simulations contains a limited (though very large) number of particles. The latent uncertainty is always present as a “hidden” component of MC dose uncertainty in simulated profiles, but our use of 2D dose difference maps allowed this MC uncertainty component to be explicitly visualized.

With the PBC, dose differences greater than ±3% are seen throughout most of the lung block. Such differences are expected with the PBC algorithm, because simple 1D density‐based corrections (e.g., ETAR, Modified Batho) are applied to account for the tissue inhomogeneities. As reported in other investigations,^(^ ^3^ ^,^ ^5^ ^,^ ^19^ ^,^ ^23^ ^,^ ^30^ ^,^ ^34^ ^,^ ^35^ ^)^ spatial extent and magnitude of the discrepancies are more severe with increasing beam quality and decreasing field size. For the highest beam energy and smallest field size combination investigated (18‐MV, 4×4 cm), dose differences as high as 16.1% were observed. Those observations indicate that, at the edge of the beam, where loss of lateral electronic equilibrium is much more pronounced in low‐density material than in water, the PBC fails to accurately model the broadening of the beam penumbra. This high value of the dose difference is within 2% of published results obtained with more standard dosimetric evaluation techniques.^(^ ^4^ ^,^ ^23^ ^,^ ^25^ ^)^

Compared with the PBC, the AAA yields dose distributions that are in better overall agreement with the MC results. Specifically, the AAA reports more accurate lung doses in the penumbra region. For the 18‐MV beam configurations, deviations greater than 4% are observed in the beam penumbra region; however, they are restricted to small regions. For the 6‐MV beams studied, deviations are, for the most part, less than 3%. However, it can be seen that AAA underestimates the dose within lung near the vertical interface by up to 4%. It also overestimates the dose in the secondary buildup region following the lung block by up to 3.5% for the 10×10‐cm field. For the 18‐MV beams, the secondary buildup and vertical interface regions were both well modeled (deviations less than 2%). During our investigation, we observed maximum deviations of AAA calculations from MC results that were larger (9.6%) than those previously reported (4%) by the developers of the algorithm.^(^ ^14^ ^,^ ^15^ ^)^ Differences in the phantom used in our investigation as compared with the phantoms previously used^(^ ^14^ ^,^ ^15^ ^)^ and modifications to the algorithm since its development^(16)^ likely explain the increased discrepancy. The much larger number of MC comparison points used in our evaluation of the AAA is also likely to be a contributing factor.

**Figure 5:**
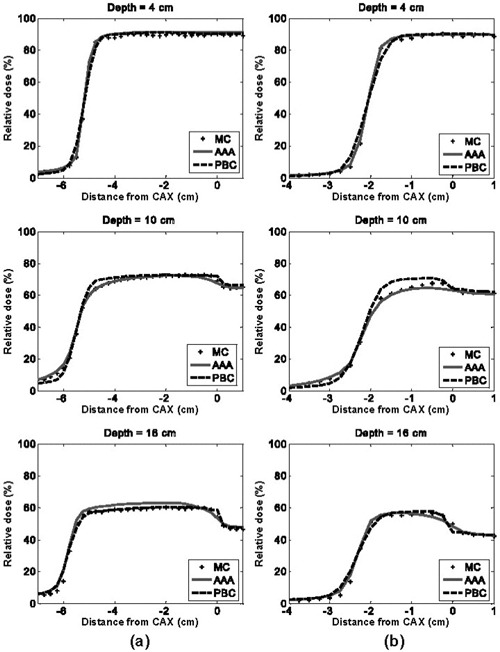
Horizontal dose profiles at various depths in region of interest A for 6‐MV, normal‐incident beam configurations, where PBC is pencil beam convolution, MC is Monte Carlo system, AAA is analytical anisotropic algorithm, and CAX is the central axis: (a) 10×10 cm, 0‐degree gantry angle; and (b) 4×4 cm, 0‐degree gantry angle

**Figure 6:**
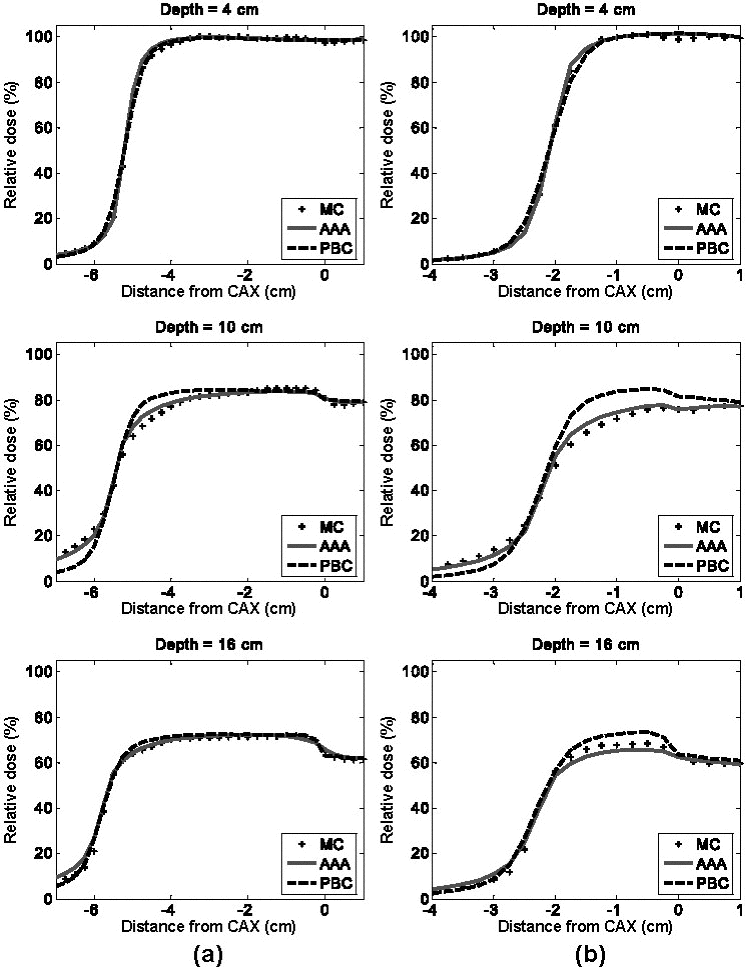
Horizontal dose profiles at various depths in region of interest A for 18‐MV, normal‐incident beam configurations, where PBC is pencil beam convolution, MC is Monte Carlo calculation, AAA is analytical anisotropic algorithm, and CAX is the central axis: (a) 10×10 cm, 0‐degree gantry angle; and (b) 4×4 cm, 0‐degree gantry angle

Figs. 5 and 6 provide off‐axis dose profiles of 6‐MV and 18‐MV beams at depths of 4 cm (just above the water–lung interface), 10 cm (across the mid‐lung), and 16 cm (immediately under the lung–water interface). These profiles more accurately quantify the differences, demonstrating that the AAA results are closer to MC in all cases except the 6‐MV 10×10‐cm field, where the AAA over‐predicted the dose to the secondary buildup region under the lung–water interface by ~3.5%.

Table 2 gives the mean value and the standard deviation of the dose differences within ROI A, a region encompassing the lung inhomogeneity and surrounding solid water, for both the 6‐MV and 18‐MV open beam configurations. A small mean value of the dose difference does not necessarily reflect better agreement, because negative and positive deviations can compensate each other, but a small standard deviation can be seen as a true indicator of overall agreement.

**Table 2:** Mean dose difference, standard deviation of the dose differences, and confidence limit within region of interest A, a region encompassing the lung block and surrounding water, for all open beam configurations, calculated with the pencil beam convolution (PBC) and the analytical anisotropic algorithm (AAA)

Beam configuration	Mean dose difference (%)		Standard deviation of the differences (%)		Confidence limit (%)	
	PBC	AAA	PBC	AAA	PBC	AAA
1	0.8	0.7	1.8	1.5	3.5	3.0
2	1.2	0.1	2.7	1.4	5.3	2.2
3	0.5	0.3	2.1	1.1	3.7	2.0
4	0.5	0.4	3.8	1.9	6.2	3.3
5	2.8	0.4	5.6	2.4	11.2	4.0
6	0.4	0.2	4.0	1.6	6.4	2.6

Table 2 also presents the confidence limits for each beam configuration. Application of a confidence limit that combines the systematic deviations and their spread into a single value is very useful for evaluating differences within large matrices of comparison points.

The statistics presented in Table 2 lead to the conclusion that, as compared with the PBC, the AAA results in more accurate dose distributions in the vertical water–lung interface phantom. For each open beam configuration, the AAA yielded smaller means, standard deviations, and confidence limits. On average, the standard deviation of the dose differences was reduced by half with the AAA dose calculation model. Confidence limits for the PBC ranged from 3.5% for the 6‐MV 10×10‐cm beam to 11.2% for the 18‐MV 4×4‐cm beam; for the AAA, the confidence limits did not exceed 4% for all beam configurations. The worst agreement of the AAA with the MC results was observed for the highest energy and smallest field combination (18 MV, 4×4 cm). However, the standard deviations of the differences (2.4%) and the confidence limit (4.0%) were right between the standard deviations and confidence limits provided by the current clinical PBC model of the 6‐MV 4×4‐cm and 10×10‐cm beams.

**Figure 7:**
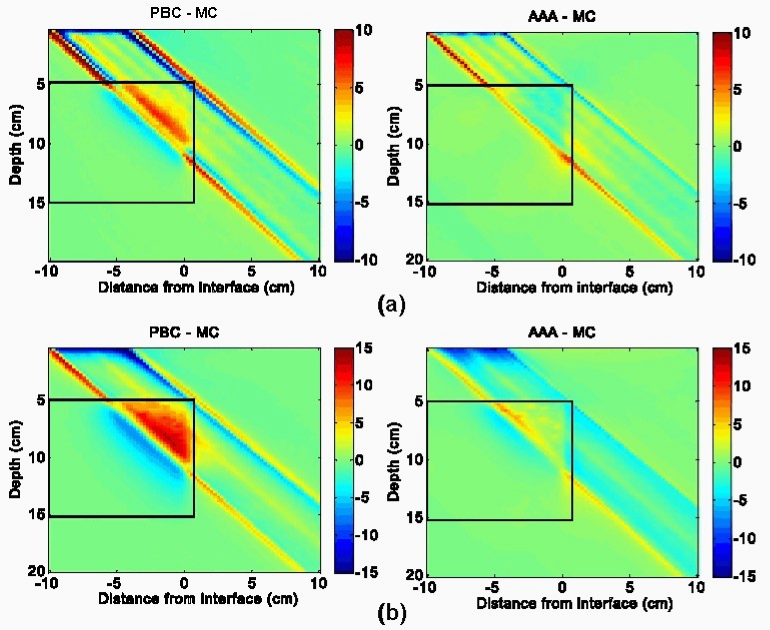
Pencil beam convolution (PBC)–Monte Carlo (MC) and analytical anisotropic algorithm (AAA)–MC dose difference maps for obliquely incident beam configurations: (a) 6‐MV beam energy, and (b) 18‐MV beam energy

**Figure 8:**
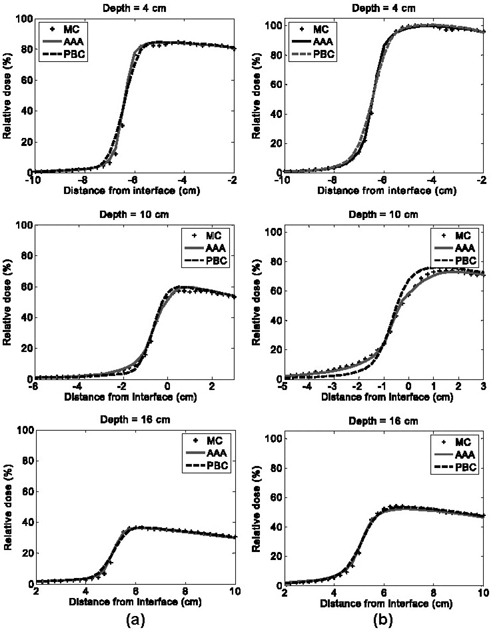
Horizontal dose profiles at various depths in region of interest A for obliquely incident beam configurations, where PBC is pencil beam convolution, MC is Monte Carlo calculation, and AAA is analytical anisotropic algorithm: (a) 6‐MV beam energy, and (b) 18‐MV beam energy

Fig. 7 shows the dose difference distributions for oblique 4×4‐cm 6‐MV and 18‐MV beams. Agreement of the PBC and the AAA with MC is slightly better for both algorithms in this case than in the case of normal beam incidence. Dose deviations in the penumbral region are smaller both in magnitude and in volume. Under‐prediction of lung doses from the 6‐MV beam with the AAA near the vertical boundary was also less in magnitude. These observations can be confirmed by comparing the profiles in Fig. 8 for oblique beam incidence with those given in Figs. 5(b) and 6(b) for normal beam incidence. The PBC and the AAA profiles are closer to the MC data in all panels of Fig. 8 than in the equivalent panels in Figs. 5 and 6. The standard deviation of the dose differences and confidence limits (see Table 2) for the oblique beams are also smaller than are those for the normal incident beams, which indicates better agreement with the MC results.

Traditionally, TPS verification involves comparison of calculated doses with data measured in both simple and complex phantoms, and this verification method remains a “gold standard.” Nowadays, MC‐generated data are gaining acceptance in TPS verifications that involve complex heterogeneous media. There are several reasons for the rising popularity of the MC method in TPS dosimetric evaluation of complex geometries. First, MC statistical uncertainties (often less than ±1% to 2% at 1 standard deviation) can now be reduced below experimental uncertainties in the thermoluminescent detector measurements (±1.5% to 2.5% at 1 standard deviation) commonly used in dose verifications involving anthropomorphic phantoms.^(^ ^4^ ^,^ ^9^ ^,^ ^12^ ^,^ ^22^ ^,^ ^23^ ^,^ ^36^ ^)^ Furthermore, uncertainties associated with imperfect positioning of detectors relative to their intended position in the phantom are eliminated with MC, because the position and size of every voxel is known exactly. Another advantage of MC is that it provides a large number of comparison points—a number considerably exceeding the number that can be accurately measured. As a result, more‐sophisticated techniques such as dose difference maps and histograms can be used in the analysis, producing a more comprehensive dosimetric evaluation of the dose calculation algorithms. However, it should be kept in mind that, because the number of comparison points in MC verification is considerably larger, deviations larger than those measured experimentally are expected. The reason is that relatively sparse experimental points are likely to miss the position where the largest error occurs.

The results shown in Figs. 3–8 and Table 2 demonstrate that, in all tested configurations, the AAA calculates dose distributions more accurately than the PBC does. That finding is expected because the considerably greater complexity of the AAA provides extra flexibility to model dose deposition in heterogeneous geometries. The AAA calculates the dose kernels “on the fly” as a function of radiologic density around the calculation point. Clearly, the capability to alter the scatter dose kernel laterally and the introduction of a variable scatter kernel in the beamlet direction manifest in improved dose modeling. Interestingly, modeling of oblique beams is slightly more accurate than modeling of normal incident ones. This effect probably arises because the lateral scatter to be corrected for is in fact less and also because transition of scattering conditions along the beamlet is smoother for the oblique beams. This property of the AAA could be beneficial in clinical situations, in which most interfaces have oblique components. Improved modeling of the buildup and builddown regions, including the secondary buildup for an 18‐MV beam, results from the combination of more accurate modeling of the in‐beamlet scatter kernel and introduction of a more complex energy fluence model. However, the model parameters may currently be suboptimal for the 6‐MV beam, which showed larger differences in the secondary buildup region.

Numerous recommendations have been made about the accuracy in dose calculations required or achievable by TPSs. In the early era of computerized treatment planning, when dose calculation algorithms and treatment plans were less complex, the simple recommendations provided made a distinction only between criteria to be applied in low dose and high dose gradient regions.^(^ ^37^ ^–^ ^40^ ^)^ In recent years, with the advent of image‐based 3D treatment planning and the use of conformal treatment planning and delivery approaches, TPSs have significantly increased in their level of sophistication and complexity. These changes have led to a broader set of acceptability criteria being applied to a larger number of regions.^(^ ^26^ ^,^ ^31^ ^,^ ^41^ ^–^ ^44^ ^)^

To illustrate the foregoing point, here is a sample set of criteria proposed by Venselaar et al.^(31)^ for an homogeneous, simple geometry:

- ±2% central beam axis
- 2 mm or ±10% in high dose, large dose gradient
- ±3% in high dose, small dose gradient
- ±3% in low dose, small dose gradient
- 2 mm radiologic width
- 2 mm beam fringe

These investigators also used various normalization conditions to generate the acceptability criteria, resulting in a range of values that are not directly comparable. Lack of comparability makes a pass/fail evaluation of a particular algorithm relative to the foregoing criteria inappropriate if a different normalization technique is used. Nevertheless, these values provide an indication of the range of accuracy that is expected and achievable in modern TPSs for a complex heterogeneous phantom, and our results show that the AAA demonstrated accuracy well within that range.

## IV. CONCLUSIONS

The AAA and the PBC were compared against Monte Carlo calculations in an EWLI phantom for 6‐MV and 18‐MV photon beams. The calculations and analyses were performed on 2D dose matrix with a voxel size of 2.5×2.5 mm. Dose difference maps and dose profiles were used to evaluate performance of the algorithms. Our results for the PBC algorithm agree with and can be considered to be a 2D extension of the study previously published by Cranmer‐Sargison et al.^(23)^ Our results also agree with data reported previously^(^ ^15^ ^,^ ^24^ ^)^ on the accuracy of AAA calculations in homogeneous media, but the heterogeneous phantoms used by Ulmer et al.^(15)^ were substantially different from our EWLI phantom, and therefore results from the two studies cannot be directly compared.

Our results show that, as compared with the PBC, the AAA models the penumbra more accurately both in water and in lung. The AAA, with its more complex accounting of heterogeneities, also provides a more accurate estimate of the dose within the lung block and surrounding water than the PBC does. In heterogeneous conditions (i.e., the EWLI phantom), AAA calculations for the small 4×4‐cm 18‐MV beam, which is poorly modeled by the PBC, provide the same accuracy as the commonly acceptable (in clinical situations) PBC model of 6‐MV beams.

Future work will include a dosimetric comparison of various commercially available superposition–convolution algorithms in our EWLI phantom and in clinical situations.

## ACKNOWLEDGMENTS

The authors gratefully acknowledge Dr. William Ansbacher for many very useful discussions on details of the PBC and the AAA.
